# Balancing Thermal Management and Wear Resistance via Tungsten-Mediated Architectural Stabilization of Cu-Based Interpenetrating Phase Composites

**DOI:** 10.3390/ma19163394

**Published:** 2026-08-10

**Authors:** Han Hu, Ziqiang Dong, Yanjie Liu, Yi Liu

**Affiliations:** 1 Materials Genome Institute & State Key Laboratory of Materials for Advanced Nuclear Energy & Shanghai Engineering Research Center for Integrated Circuits and Advanced Display Materials, Shanghai University, Shanghai 200444, China; hanhu0526@shu.edu.cn; 2 School of Mechanical and Energy Engineering, Shanghai Technical Institute of Electronics & Information, Shanghai 201411, China; 3 Institute of Low-Dimensional Carbons and Device Physics, Shanghai University, Shanghai 200444, China

**Keywords:** Cu-based interpenetrating phase composites, W-mediated skeleton regulation, thermo-tribological response, electrical-thermal-wear balance, pressureless infiltration

## Abstract

Cu-based interpenetrating phase composites (IPCs) are candidate materials for sliding-contact applications in which thermal management, wear resistance, and electrical transport must be balanced. Herein, Cu–(CrW*_x_*)C IPCs were fabricated by pressureless infiltration using nominal W-addition indices *x* = 0, 10, 25, and 50, corresponding to 0, 10, 25, and 50 g W added per 100 g Cr_2_O_3_ rather than final W mass fractions. Microstructural characterization indicates that W addition changes the scale and connectivity of W-containing carbide regions while preserving a continuous Cu-rich network. Representative quasi-static compression curves illustrate the large-strain load-bearing response but are interpreted descriptively because independent replicate specimens were not available for every composition under an identical test matrix. All composites retain room-temperature electrical conductivities of 39.23–40.44% of the International Annealed Copper Standard (IACS). Cu–(CrW10)C reaches a thermal conductivity of 208.27 W m^−1^ K^−1^ at 500 °C and the lowest specific wear rate of 2.42 × 10^−6^ mm^3^ N^−1^ m^−1^. Worn-surface and cross-sectional observations show that its low material loss coexists with localized cracking and adhered/detached features, whereas higher nominal W additions promote carbide fragmentation, interfacial separation, and hard-debris-mediated abrasion. The thermo-tribological performance index (TTPI) and electrical-thermal-wear balance index (ETWBI), used as internal screening metrics, identify Cu–(CrW10)C as the most balanced composition within the present four-composition dataset. These results demonstrate that wear resistance is governed by the stability of the skeleton-supported tribologically modified layer and continuous Cu-network transport rather than by hardness or nominal W addition alone.

## 1. Introduction

Sliding-contact components used in electrical contacts, thermal management units, brake/friction pairs, and other moving assemblies rarely fail through purely mechanical or purely thermal processes. During sliding, asperity ploughing, adhesive shearing, tribo-oxidation, and debris generation occur concurrently with friction-induced temperature rise. The resulting heat can accelerate surface softening, oxidation, interfacial degradation, and thermal stress accumulation, while continuous material removal changes the real contact area, load transfer path, and heat dissipation condition. Therefore, materials intended for such environments should not be evaluated solely by friction coefficient, hardness, or wear rate. A more relevant design criterion is the simultaneous control of material removal, heat dissipation, and thermal dimensional stability, with electrical transport also remaining critical for conductive sliding contact applications [1,2].

This requirement creates an intrinsic materials–design trade-off. Hard ceramics and refractory carbides resist asperity penetration and abrasive ploughing, but their limited fracture toughness and poor damage accommodation can lead to brittle cracking, unstable hard debris, and local delamination during repeated sliding. By contrast, Cu and Cu-based alloys provide high electrical and thermal conductivity together with plastic accommodation; however, their low hardness and limited resistance to abrasive wear restrict their direct use under severe contact conditions. Particle-reinforced Cu composites partly mitigate this problem, yet isolated reinforcements and discontinuous metallic pathways often hinder the simultaneous realization of stable load bearing, continuous transport, and effective crack/debris control [3,4,5,6,7,8,9,10].

Interpenetrating phase composites (IPCs) provide a more suitable structural solution because both constituent phases are spatially continuous. In Cu-based IPCs, a continuous ceramic skeleton can support the contact load and resist ploughing, whereas a continuous Cu-rich phase can provide heat/electrical transport, plastic accommodation, and crack blunting. This dual-network architecture differs fundamentally from conventional particle-reinforced composites, because load transfer, thermal conduction, and damage accommodation can all proceed through connected pathways. Consequently, the performance of Cu-based IPCs is governed not only by the intrinsic properties of the ceramic and metallic phases, but also by skeleton topology, network connectivity, and local chemical heterogeneity [11,12,13,14,15,16,17,18].

For Cr–C carbide/Cu IPCs, the ceramic skeleton is not simply a passive hard framework; it determines whether the worn surface remains supported or evolves into a source of hard third-body debris. If the skeleton is too weak or poorly connected, it cannot effectively protect the Cu-rich phase from severe ploughing and material removal. Conversely, if the skeleton is excessively hard, brittle, or compositionally heterogeneous, local stress concentration, interfacial debonding, and carbide fragmentation may be promoted. W-containing carbides offer an effective route for skeleton regulation because W can modify the local carbide constitution and morphology of the Cr–C framework. However, W addition should not be considered a monotonic hardening strategy. Moderate W-mediated regulation may stabilize the skeleton-supported tribo-damaged layer while preserving Cu-network transport, whereas excessive W-rich carbide enrichment may intensify fragmentation and third-body abrasion [19,20,21,22,23,24].

Although Cu-based IPCs are promising for conductive, thermally dissipative, and wear-resistant components, the coupling among skeleton regulation, electrical/thermal transport, thermal expansion, and wear damage remains insufficiently understood. Many studies evaluate hardness, friction coefficient, wear rate, electrical conductivity, or thermal conductivity as separate properties. Such single-property assessment can be misleading for sliding contact materials: a low friction coefficient does not necessarily guarantee low volume loss when the tribo-damaged layer is unstable, and a hard material may still wear rapidly if hard fragments continuously generate third-body abrasives. Likewise, a low wear rate alone is insufficient when frictional heat cannot be dissipated or thermal expansion induces dimensional instability. Complementary materials development studies have shown that data-driven high-throughput experiments can accelerate mapping of multicomponent composition–property relationships, while micron-grain/nano-twin engineering can strongly influence electrical transport in Cu [25,26]. More recent materials–informatics research has further shown that non-linear relationships and inter-feature effects should be evaluated explicitly when constructing property prediction models for multi-component alloys [27]. A coupled evaluation framework is therefore needed to identify compositions that balance skeleton-supported wear resistance with Cu-network transport and thermal-expansion constraint.

In this work, Cu–(CrW*_x_*)C IPCs with nominal W-addition indices *x* = 0, 10, 25, and 50 were fabricated by infiltrating Cu into porous Cr–W–C carbide preforms. Here, *x* denotes the mass of W powder added per 100 g Cr_2_O_3_ and does not represent the final W weight percentage (wt.%) of the preform or IPC. Their phase constitution, preform morphology, interpenetrating architecture, representative quasi-static compressive response, thermophysical and electrical properties, hardness, friction behavior, wear volume, worn-surface chemistry, and subsurface damage were systematically investigated. A thermo-tribological performance index (TTPI) was used to correlate specific wear rate, thermal conductivity, and thermal expansion, while an electrical-thermal-wear balance index (ETWBI) additionally incorporated electrical conductivity. Both indices are used only for internal comparison of the four compositions. The objective was to reveal how W-mediated carbide-skeleton regulation controls the balance between skeleton-supported wear resistance and Cu-network transport.

## 2. Experimental Procedure

### 2.1. Preparation of (CrW_x_)C Preforms and Cu–(CrW_x_)C IPCs

Commercial Cr_2_O_3_ powder (median particle diameter (*D*_50_) ≈ 10 *μ*m; Sichuan Minghong Hengjin Technology Co., Ltd., Mianyang, China), W powder (99.95 wt.%, 1–3 *μ*m; Adamas Reagent Co., Shanghai, China), and Cu powder (99.9 wt.%, 325 mesh; Adamas Reagent Co., Shanghai, China) were used as starting materials. Based on 100 g of Cr_2_O_3_, 0, 10, 25, or 50 g of W powder was added to prepare four porous carbide preform compositions, denoted as (CrW0)C, (CrW10)C, (CrW25)C, and (CrW50)C, respectively. The Cr_2_O_3_ and W powders were mixed in zirconia jars with zirconia balls using absolute ethanol as the milling medium. Ball milling was performed at 150 revolutions per minute (rpm) for 12 h with a ball-to-powder ratio of 5:1 and a ball–size ratio of 5 mm:10 mm:20 mm = 1:1:1, followed by forced-air drying at 60 °C for 12 h.

In this notation, *x* is a nominal batch index equal to the grams of W powder added per 100 g Cr_2_O_3_. It is not the W mass fraction of the starting powder mixture, reduced carbide preform, or final Cu-infiltrated composite. The nominal W/Cr atomic ratios calculated from the starting batches are 0, 0.0413, 0.1033, and 0.2067 for *x* = 0, 10, 25, and 50, respectively. Under the idealized assumption of complete conversion to Cr_3_C_2_- and W-containing carbide products, the estimated W contents in the final IPCs are 0, 4.31, 9.85, and 17.10 wt.%, respectively. These calculation-based values define the nominal design levels and are not treated as measured local compositions or quantitative phase fractions.

To assess whether the composition-dependent transport and wear responses could be explained primarily by different amounts of infiltrated Cu, the bulk Cu-phase volume fraction was estimated using the measured preform porosity together with the Archimedes bulk density balance of the infiltrated IPCs. The calculation assumes that the accessible pore volume is occupied by Cu and that the remaining solid volume belongs to the carbide skeleton. The estimated Cu fractions for Cu–(CrW0)C, Cu–(CrW10)C, Cu–(CrW25)C, and Cu–(CrW50)C are 56.13, 56.08, 56.84, and 55.68 volume percent (vol.%), respectively. These values are approximate bulk phase fractions rather than direct three-dimensional segmentation results; residual closed porosity, local composition heterogeneity, and the density adopted for the carbide skeleton are potential sources of uncertainty.

The dried powders were uniaxially pressed at 100 MPa. Disk preforms with nominal dimensions of φ20 × 5 mm were held for 12 h during carbothermal reduction, whereas bar preforms with nominal dimensions of 50 × 10 × 10 mm were held for 20 h to accommodate the larger characteristic gas transport and reaction distance. Carbothermal reduction was performed at 1150 °C using a heating rate of 5 °C min^−1^ under CH_4_/H_2_/Ar gas flow rates of 40/180/180 standard cubic centimeters per minute (sccm), respectively. After carbothermal reduction, the atmosphere was switched to H_2_ containing approximately 150 parts per million (ppm) H2O at 500 sccm for 3–5 h to remove residual free carbon and improve the interfacial condition for Cu infiltration.

Pressureless Cu infiltration was then conducted at 1250 °C for 5 h in dry H_2_ (200 sccm) using BN-coated alumina crucibles. The Cu source was placed beneath the porous carbide preform so that molten Cu spontaneously infiltrated upward into the open carbide skeleton. After furnace cooling, the infiltrated IPCs were ground, polished, and machined into the required specimens. The final composites are denoted as Cu–(CrW0)C, Cu–(CrW10)C, Cu–(CrW25)C, and Cu–(CrW50)C.

### 2.2. Phase and Microstructure Characterization

**Figure 1:**
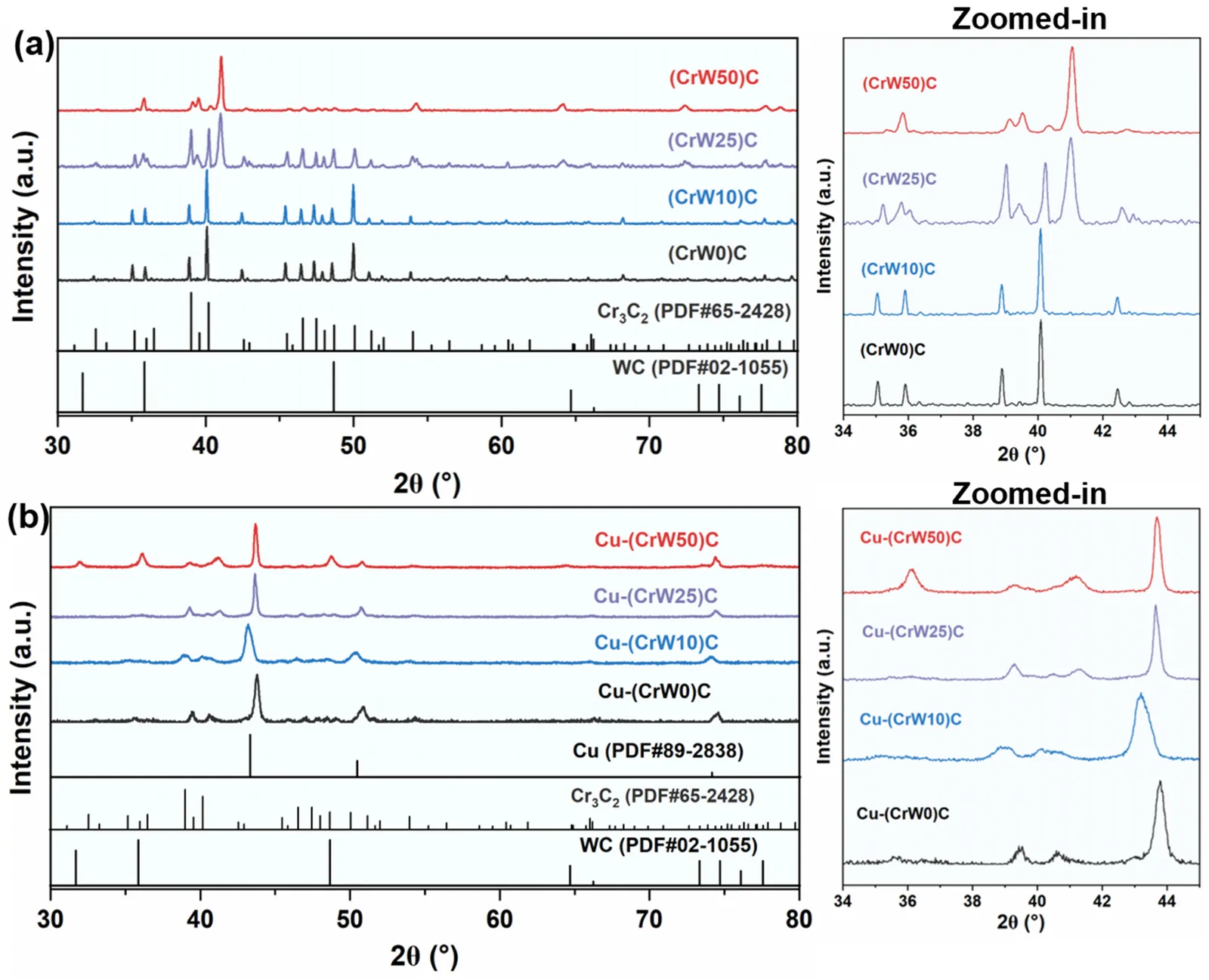
X-ray diffraction (XRD) patterns of (CrW*_x_*)C preforms and Cu–(CrW*_x_*)C interpenetrating phase composites (IPCs): (**a**) preform patterns and enlarged carbide-reflection region; (**b**) Cu-infiltrated IPC patterns and corresponding enlarged region. The common 30–80° range is shown for direct comparison. The same Cr_3_C_2_ and tungsten carbide (WC) reference entries from the Powder Diffraction File (PDF; PDF#65-2428 and PDF#02-1055, respectively) are used in both panels; no horizontal peak correction was applied. The WC reference is included only for comparison and is not treated as evidence for identification of an independent WC phase.

The phase constitution of the porous preforms and Cu-infiltrated IPCs was characterized by X-ray diffraction (XRD; Bruker D2, Bruker AXS SE, Ettlingen, Germany) using Cu Kα radiation (*λ* = 1.5406 Å). The preforms were scanned over 2*θ* = 30–110°, whereas the Cu-infiltrated IPCs were scanned over 2*θ* = 30–80°, with a step size of 0.02° and a scan rate of 2° min^−1^. Figure 1 displays the common 30–80° range for direct comparison. No horizontal peak shift or post-acquisition peak-position correction was applied. The same Cr3C2 and WC reference entries from the Powder Diffraction File (PDF; PDF#65-2428 and PDF#02-1055, respectively) were used in both panels; Cu was compared with PDF#89-2838. Reference cards were used only for qualitative phase identification and comparison. The WC reference is included only for comparison and is not treated as evidence for identification of an independent WC phase.

The morphology and elemental distribution of the porous preforms and IPCs were examined by field-emission scanning electron microscopy (SEM; Gemini 300, Carl Zeiss AG, Oberkochen, Germany) equipped with energy-dispersive X-ray spectroscopy (EDS; Ultim Max 65, Oxford Instruments, Abingdon, UK). Cross-sectional IPC specimens were ground, polished, and ultrasonically cleaned before observation. Back-scattered electron contrast was used to distinguish the Cu-rich phase and carbide regions; composition-specific labels were assigned only when supported by location-matched EDS. Worn-surface maps for the four compositions were acquired and displayed using identical operating and intensity-scaling conditions, allowing qualitative comparison of spatial distributions but not direct conversion of map brightness to concentration. EDS point-analysis values are reported in atomic percent (at.%) in Table S1. Carbon values are treated as semiquantitative and are not used to determine exact carbide stoichiometry.

### 2.3. Thermophysical and Electrical Characterization

Thermal diffusivity was measured at 25, 300, and 500 °C using a laser flash analyzer (NETZSCH LFA 467, NETZSCH-Gerätebau GmbH, Selb, Germany) under an N_2_ purge/protective atmosphere. Specimens for laser flash analysis were machined into plates with nominal dimensions of 10 mm × 10 mm × 2 mm. Before testing, the specimen surfaces were ground and polished without graphite coating. Room-temperature bulk density was determined by the Archimedes method and used both for thermal conductivity calculation and, together with the measured preform porosity, for estimating the bulk Cu-phase volume fraction. At each temperature, three shots were collected and analyzed using the standard model with pulse correction. The laser voltage and pulse width were 250 V and 0.40 ms, respectively. Thermal conductivity was calculated from the measured thermal diffusivity, heat capacity, and density according to [28].

(1) $$\lambda (T)=D_{\mathrm{th}}(T)C_{p}(T)\rho (T)$$

where *λ*(*T*), *D*_th_(*T*), *C*_p_(*T*), and *ρ*(*T*) denote thermal conductivity, thermal diffusivity, heat capacity, and density at temperature *T*, respectively. At each temperature, three repeated laser-flash shots were collected on one specimen per composition. The main-text values are reported as mean ± sample standard deviation (SD), while the root-mean-square deviation (RMSD) about the three-shot mean is provided in Table S2 of the Supplementary Materials; these quantities describe repeated-shot measurement dispersion rather than specimen-to-specimen variability. Thermal expansion was measured using a DIL 402 C dilatometer (NETZSCH-Gerätebau GmbH, Selb, Germany) under Ar flowing at 80 mL min^−1^. One cylindrical specimen per composition (nominally φ3 mm × 10 mm) was heated at 10 °C min^−1^. The thermal expansion curves were referenced to 30 °C, and the average coefficient of thermal expansion (CTE) from 30 to 500 °C was calculated from Δ*L*/*L*_0_. Because one specimen was measured per composition, no specimen-to-specimen CTE error bar is claimed.

(2) $$\alpha_{30–500}=\frac{\Delta L/L_{0}}{500–30}$$

where *L*_0_ is the specimen length at 30 °C and Δ*L* = *L*(500 °C) − *L*_0_.

Room temperature electrical conductivity was measured at 20 °C using an eddy current conductivity meter (FD102, Xiamen First Electronic Technology Co., Ltd., Xiamen, China) at 120 kHz. The instrument was calibrated with Al and pure Cu reference blocks before measurement. Ten spatially separated positions were measured on the polished surface of one specimen per composition, and the values are reported as mean ± sample standard deviation, expressed as a percentage of the International Annealed Copper Standard (IACS). The error bars therefore describe within-specimen spatial variability rather than specimen-to-specimen variability. For the transport consistency analysis, the conductivity measured at 20 °C was corrected to 30 °C and used to estimate electronic thermal conductivity through the Wiedemann–Franz relation [29].

### 2.4. Hardness, Quasi-Static Compression, Tribological Testing and Wear Analysis

Vickers microhardness was measured using a Qness 60 A+ EVO hardness tester (ATM Qness GmbH, Golling, Austria) under a 0.1 kgf test force (HV0.1), with a dwell time of 15 s. Ten valid spatially separated indentations were collected on one specimen per composition. Results are reported as mean ± sample standard deviation, which describes within-specimen spatial variability. Before tribological testing, specimens were ground, polished, and ultrasonically cleaned to ensure comparable initial surface conditions.

Quasi-static compression tests were performed at room temperature using a ZwickRoell Z100 universal testing machine (ZwickRoell GmbH & Co. KG, Ulm, Germany) on cylindrical IPC specimens with a nominal diameter of approximately 3 mm and a height of approximately 6 mm at an engineering strain rate of 0.001 s^−1^. Engineering stress and strain were calculated from the measured initial dimensions and recorded load displacement data. The curves are presented as representative large strain responses. Because independent replicate specimens from the same batch and identical test matrix were not available for every composition, numerical differences are interpreted descriptively and are not treated as statistically resolved.

Ball-on-disk sliding wear tests were conducted using a UMT-Tribolab tribometer (Bruker Corporation, San Jose, CA, USA) and a Si_3_N_4_ counter ball under a normal load of 12 N. The rotation speed was 200 r min^−1^, the wear-track radius was 7 mm, and the actual sliding time was 1202 s, corresponding to a sliding distance of 176.22 m. The coefficient of friction (COF) was recorded continuously. Because several curves underwent pronounced transitions after 250 s, the arithmetic mean over 250–1202 s is reported as a fixed post-running-in comparison value rather than a strictly stationary steady-state coefficient. The present test is treated as a short-duration sliding–screening experiment.

After testing, the worn surfaces and cross sections were characterized by SEM/EDS. Three-dimensional wear morphology was measured using a ContourX-100 optical profilometer (Bruker Corporation, Tucson, AZ, USA), and wear volume was obtained by integrating the wear scar profile over the circular wear track. The measured wear volume was then used to calculate the specific wear rate and the thermo-tribological coupling indicators described below.

### 2.5. Thermo-Tribological Coupling Calculation

To quantify the thermo-tribological coupling response, frictional energy input, material removal, heat-dissipation capability, and thermal expansion tendency were correlated using the following equations. The linear sliding speed and sliding distance were calculated as

(3) $$v=\frac{2\pi rn}{60}$$

(4) $$S=vt=\frac{2\pi rnt}{60}$$

where *r* is the wear track radius, *n* is the rotation speed, and *t* is the sliding time. The specific wear rate was calculated according to Archard’s relation [30] as

(5) $$K=\frac{V}{F_{N}S}$$

where *V* is the wear volume and *F*_N_ is the normal load. Frictional work and frictional heat-generation power were estimated using the average friction coefficient, *μ*_avg_:

(6) $$W_{f}=\mu_{\mathrm{avg}}F_{N}S$$

(7) $$Q_{f}=\mu_{\mathrm{avg}}F_{N}v$$

The wear loss per unit frictional work was defined as

(8) $$K_{E}=\frac{V}{W_{f}}$$

The relative heat accumulation tendency and thermal stress tendency were described as

(9) $$H=\frac{Q_{f}}{\lambda_{500}}$$

(10) $$\Pi_{\mathrm{th}}=\frac{\alpha_{30–500}Q_{f}}{\lambda_{500}}$$

Finally, the thermo-tribological performance index (TTPI) and normalized TTPI were defined as

(11) $$\mathrm{TTPI}=\frac{\lambda_{500}}{K\alpha_{30–500}}$$

(12) $${\mathrm{TTPI}}_{\mathrm{norm}}=\frac{{\mathrm{TTPI}}_{i}}{{\mathrm{TTPI}}_{\mathrm{Cu}–(\mathrm{CrW}10)C}}$$

A higher TTPI represents a better balance among low wear rate, high thermal conductivity, and reduced thermal-expansion-induced instability. In this work, TTPI values were normalized to that of Cu–(CrW10)C to enable direct comparison among the four IPC compositions.

To further evaluate the coupled electrical-thermal-wear balance of the present IPCs, an electrical-thermal-wear balance index (ETWBI) was introduced. For the *i*-th composition, the normalized thermal conductivity, electrical conductivity, wear rate and thermal expansion terms were defined as follows. The favorable thermal conductivity and electrical conductivity terms were normalized by their maximum values, whereas the wear rate and thermal expansion terms were normalized in an inverse manner because lower wear rate and lower thermal expansion are beneficial:

(13) $$\lambda_{n,i}=\frac{\lambda_{500,i}}{\max\left(\lambda_{500}\right)}$$

(14) $$\sigma_{n,i}=\frac{\sigma_{i}}{\max\left(\sigma \right)}$$

(15) $$K_{n,i}=\frac{\min\left(K\right)}{K_{i}}$$

(16) $$\alpha_{n,i}=\frac{\min\left(\alpha_{30–500}\right)}{\alpha_{30–500,i}}$$

where the subscript n denotes a normalized quantity, and *λ*_500_, *σ*, *K*, and *α*_30–500_ are the thermal conductivity at 500 °C, room-temperature electrical conductivity, specific wear rate, and average coefficient of thermal expansion from 30 to 500 °C, respectively.

The ETWBI was then defined as the geometric mean of the four normalized terms:

(17) $${\mathrm{ETWBI}}_{i}={\left(\lambda_{n,i}\cdot \sigma_{n,i}\cdot K_{n,i}\cdot \alpha_{n,i}\right)}^{1/4}$$

The geometric mean form penalizes any severely deficient property and is therefore suitable for assessing balance rather than single-property maximization. A higher ETWBI indicates a better simultaneous balance among high-temperature heat dissipation, Cu-network electrical transport, wear resistance and thermal-dimensional stability.

TTPI and ETWBI are internal screening metrics rather than universal material constants. Their numerical values depend on the selected compositions, normalization bounds, test conditions, included variables, and equal weighting of the terms. Extension to other IPCs or multifunctional materials requires comparable measurements and application-specific selection or weighting of the performance terms.

## 3. Results and Discussion

### 3.1. Phase Constitution of Preforms and IPCs

The XRD patterns of the (CrW*_x_*)C preforms and corresponding Cu–(CrW*_x_*)C IPCs are shown in Figure 1. The (CrW0)C preform is dominated by Cr_3_C_2_-related reflections, indicating formation of a Cr–C carbide skeleton. The principal peaks of the 0 W and 10 W preforms near approximately 35.05°, 35.90°, 38.88°, and 40.08° do not show a resolvable systematic displacement in the plotting data; therefore, the low-W patterns are not used as direct proof of a homogeneous (Cr,W)_3_C_2_ solid solution.

For the 25 W preform, the 39–41° interval contains features near approximately 39.03°, 39.43°, 40.22°, and 41.01°, whereas the corresponding 50 W features occur near approximately 39.13°, 39.53°, 40.34°, and 41.05°. This interval therefore develops a reconstructed and overlapping multi-peak profile within a densely populated Cr_3_C_2_-type diffraction region rather than a single uniformly shifted reflection. Published XRD studies demonstrate that W can substitute for Cr in the orthorhombic Cr_3_C_2_ lattice and form (Cr,W)_3_C_2_-type solid solutions. Jin et al. explicitly indexed (Cr,W)_3_C_2_ using PDF 65-0897 and 35-0804 [31]. Janka et al. identified the Cr_3_C_2_ (121) and (230) reflections in the 38–45° interval and used the associated lattice-parameter changes to estimate *x* ≈ 0.04–0.08 in (Cr_1−*x*_W*_x_*)_3_C_2_ [21]. Matthews and O’Neil further reported that the Cr_3_C_2_ *a*-axis increased from the 0.5530 nm pure-phase reference to approximately 0.5544–0.5576 nm in W-containing systems and expressed the corresponding substitution using a “W_3_C_2_-equivalent” compositional calibration [32]. In that usage, “W_3_C_2_” denotes the amount of W substituting into the Cr_3_C_2_ lattice and is not an independently identified W_3_C_2_ phase. Importantly, no set of reflections in the present laboratory patterns uniquely requires assignment to an independent WC phase.

Taken together, these reports support interpreting the present 39–41° group as overlapping reflections associated primarily with compositionally heterogeneous W-substituted Cr_3_C_2_-type regions, i.e., regions compatible with a (Cr,W)_3_C_2_ solid-solution description. Rietveld studies of WC–Cr_3_C_2_–Ni systems also show that the 35–45° interval can contain strongly overlapping contributions from Cr_3_C_2_-type carbides, W_2_C-related subcarbides, oxides, and binder phases [32,33,34]. No set of reflections in the present patterns uniquely requires assignment to an independent WC phase. Contributions from other W-containing carbide environments cannot be completely excluded, but they cannot be specifically identified from the present laboratory data without an internal standard, peak deconvolution, or full-pattern quantitative refinement. Accordingly, the WC reference card is used only for qualitative comparison and is not treated as evidence of WC formation, a WC phase fraction, or a unique additional phase.

After Cu infiltration, distinct Cu reflections appear while the carbide-skeleton reflections are retained, confirming that the Cr–W–C skeleton persists during the 1250 °C infiltration treatment. No independent WC phase is identified in either the preforms or the Cu-infiltrated IPCs. Differences in the visibility and relative intensity of the overlapping W-containing Cr_3_C_2_-type reflections may arise because Cu filling changes the relative diffracting volume of the exposed skeleton, X-ray absorption and background conditions, preferred orientation, and the relative contrast between metallic and carbide peaks. In addition, cooling of the Cu-filled interpenetrating architecture may affect carbide crystallinity, local redistribution, peak width, and thermomechanical constraint. These differences are interpreted qualitatively and are not taken as evidence of WC formation or a quantitative change in WC phase fraction. No obvious Cu–Cr or Cu–W intermetallic reflections are detected.

Cu–(CrW10)C also exhibits an apparent lower-angle displacement of the Cu(111), Cu(200), and Cu(220) reflections, located at approximately 43.231°, 50.340°, and 74.102°, respectively, compared with 43.67–43.78°, 50.75–50.81°, and 74.43–74.48° for the other IPCs. Cooling-induced thermal-expansion mismatch between the continuous Cu phase and the carbide skeleton may contribute phase-specific residual strain, and differences in skeleton topology may modify its magnitude. However, the absolute offsets decrease with increasing 2*θ* (approximately 0.49°, 0.44°, and 0.35°), which is inconsistent with a uniform Cu-lattice strain and is more characteristic of specimen displacement or sample height variation. The shift is therefore reported but is not interpreted as evidence of Cu solid-solution formation or quantitatively assigned to residual stress.

### 3.2. W-Mediated Morphology Evolution of Porous Carbide Preforms

**Figure 2:**
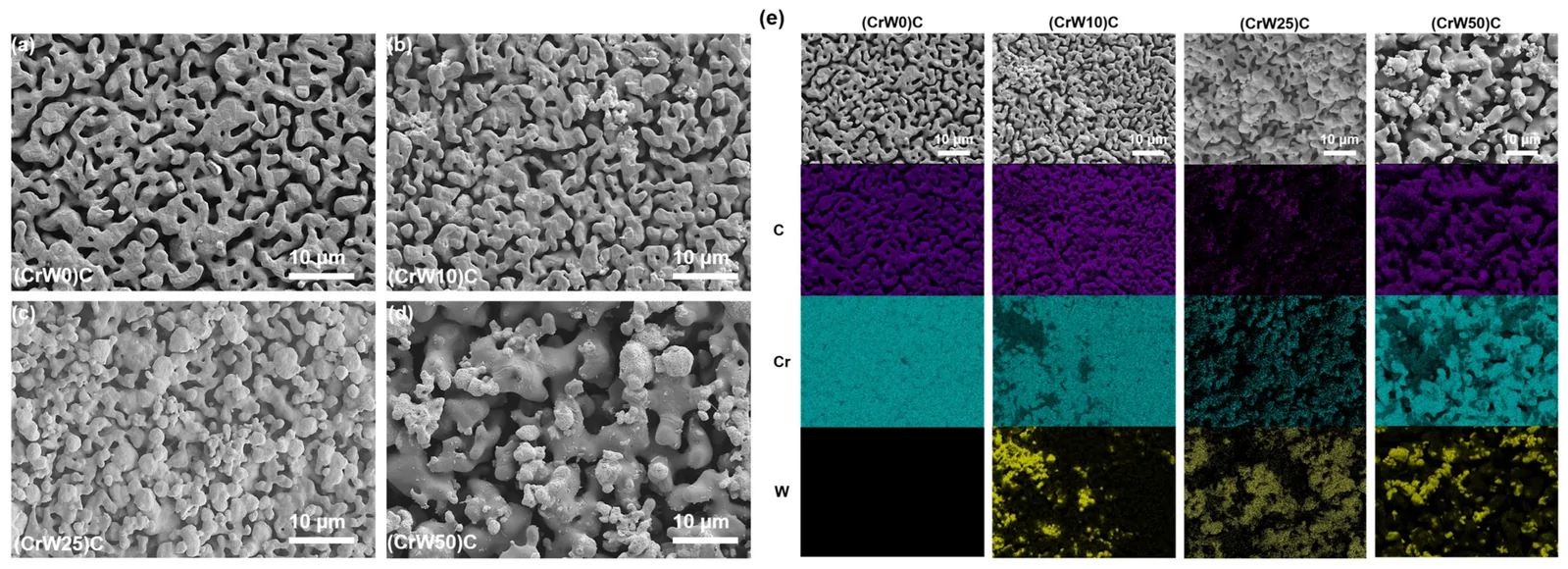
Scanning electron microscopy (SEM) images and energy-dispersive X-ray spectroscopy (EDS) elemental maps of (CrW*_x_*)C preforms. (**a**) (CrW0)C, (**b**) (CrW10)C, (**c**) (CrW25)C, and (**d**) (CrW50)C preform SEM images; (**e**) the corresponding reference SEM images and EDS maps, with sample columns in the same composition order and elemental rows labeled C, Cr, and W. Map intensity is interpreted qualitatively.

The SEM images and EDS maps of the porous preforms are shown in Figure 2. All preforms exhibit an open, interconnected skeleton architecture. The (CrW0)C preform shows a relatively continuous Cr-C carbide network. At low nominal W addition, W-containing features are fine and spatially isolated; at intermediate addition, localized coarsening and aggregation become more apparent; at the highest addition, the W-containing regions display a more connected morphology. The maps are used to identify spatial localization and morphology only; apparent brightness is not converted into concentration or used to rank the compositions.

### 3.3. Interpenetrating Architecture After Cu Infiltration

**Figure 3:**
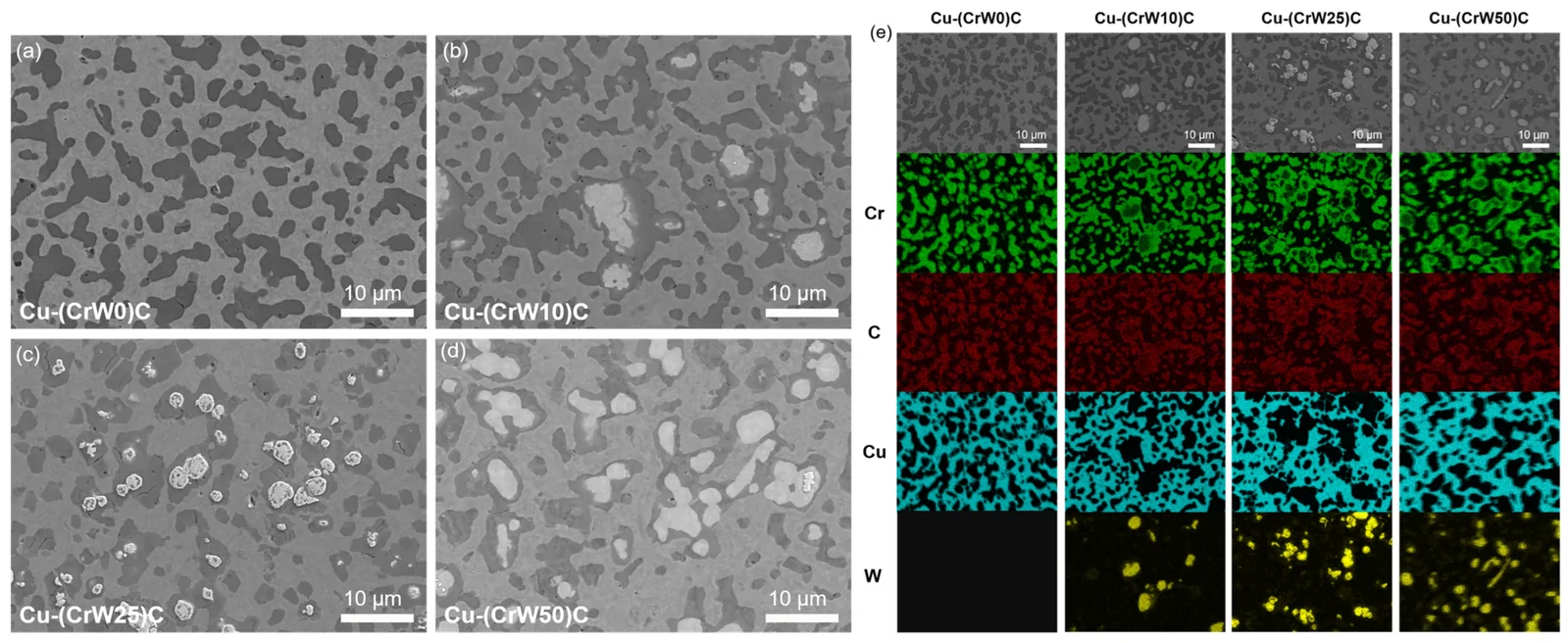
SEM images and EDS elemental maps of Cu–(CrW*_x_*)C IPCs. (**a**) Cu–(CrW0)C, (**b**) Cu–(CrW10)C, (**c**) Cu–(CrW25)C, and (**d**) Cu–(CrW50)C IPC SEM images; (**e**) the corresponding reference SEM images and EDS maps, with sample columns in the same composition order and elemental rows labeled C, Cr, Cu, and W. The maps are interpreted qualitatively.

Figure 3 shows the SEM images and EDS maps of the Cu–(CrW*_x_*)C IPCs. The Cu-rich phase occupies the original open pores and forms a continuous metallic network interpenetrating the Cr–W–C carbide skeleton. The complementary Cu and Cr/C distributions support the dual-continuous architecture at the examined scale. Cu–(CrW10)C retains a continuous Cu pathway with fine, spatially dispersed W-containing features. Cu–(CrW25)C contains locally coarsened W-containing regions, whereas Cu–(CrW50)C exhibits a more connected W-containing morphology and a more tortuous Cu pathway. The maps do not provide quantitative local W/Cr ratios or phase fractions. The density/porosity-based Cu-phase fractions are 56.13, 56.08, 56.84, and 55.68 vol.% for Cu–(CrW0)C, Cu–(CrW10)C, Cu–(CrW25)C, and Cu–(CrW50)C, respectively. The total spread is only 1.16 percentage points and shows no monotonic dependence on nominal W addition. Therefore, the principal differences in transport and wear response should be attributed primarily to skeleton topology, local carbide constitution, Cu-path tortuosity, and interface/damage evolution rather than to a large change in the total amount of Cu.

### 3.4. Quasi-Static Compressive Response

**Figure 4:**
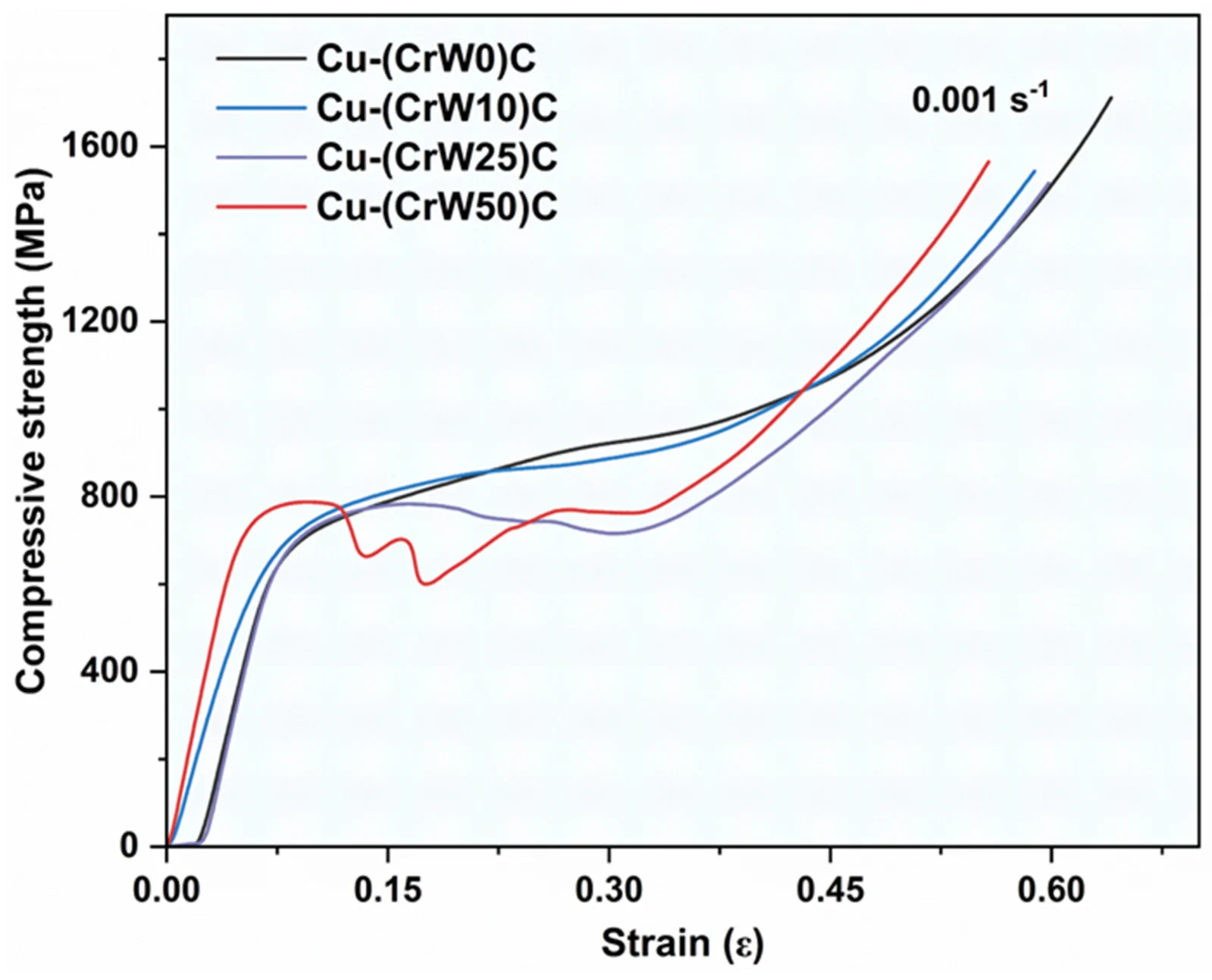
Representative quasi-static compressive stress–strain curves of Cu–(CrW*_x_*)C IPCs at 0.001 s^−1^. The curves illustrate characteristic large-strain deformation stages and are not used for statistically resolved inter-composition ranking.

The representative quasi-static compression curves at 0.001 s^−1^ are shown in Figure 4. All four IPCs exhibit rapid initial loading, a broad damage/redistribution interval, and subsequent high-strain load bearing. The maximum recorded engineering stresses for Cu–(CrW0)C, Cu–(CrW10)C, Cu–(CrW25)C, and Cu–(CrW50)C are 1711, 1544, 1516, and 1565 MPa, respectively. At a common engineering strain of 0.5, the corresponding stresses are 1177, 1201, 1157, and 1295 MPa, and the integrated energy-absorption densities U_0.5_ are 406.0, 413.8, 364.0, and 391.8 MJ m^−3^. These values are used descriptively because independent replicate curves were not available for every composition under the identical test matrix. The stress fluctuations in the 50 W curve are consistent with staged skeleton fragmentation and load-path redistribution, but small inter-composition differences are not treated as statistically significant.

### 3.5. Thermophysical and Electrical Properties

**Figure 5:**
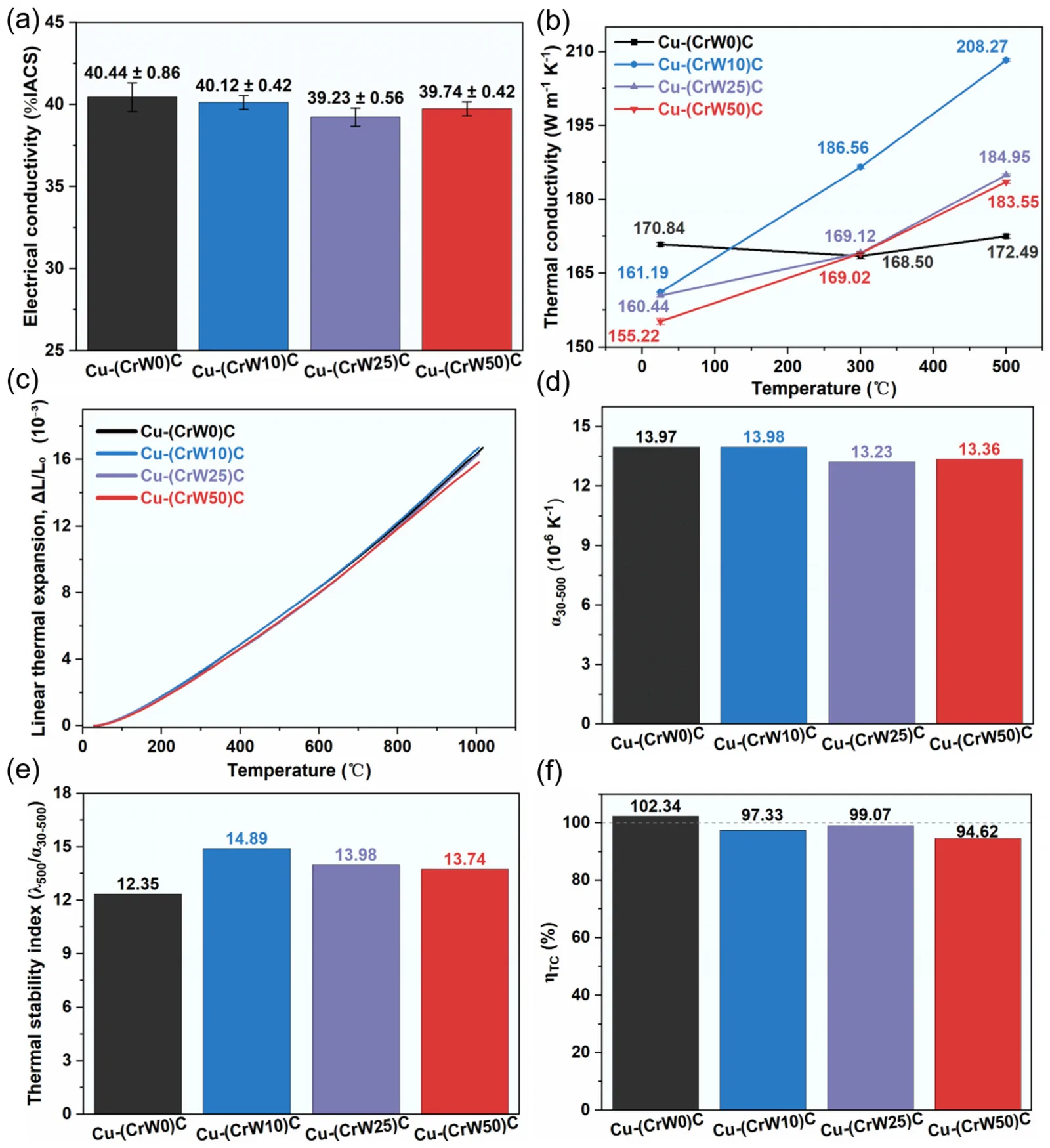
Thermophysical and electrical properties of Cu–(CrW*_x_*)C IPCs: (**a**) electrical conductivity, expressed as a percentage of the International Annealed Copper Standard (IACS), at 20 °C (mean ± standard deviation (SD) of ten spatial measurements on one specimen); (**b**) thermal conductivity at 25, 300, and 500 °C (mean ± SD of three repeated shots on one specimen per temperature); (**c**) thermal-expansion curves referenced to 30 °C; (**d**) average coefficient of thermal expansion (CTE) from 30 to 500 °C; (**e**) *λ*_500_/*α*_30–500_; and (**f**) transport-consistency factor *η*_TC_. Thermal conductivity root-mean-square deviation (RMSD) values are listed in Table S2.

The thermophysical and electrical properties are summarized in Figure 5. The room-temperature electrical conductivities of Cu–(CrW0)C, Cu–(CrW10)C, Cu–(CrW25)C, and Cu–(CrW50)C are 40.44 ± 0.86, 40.12 ± 0.42, 39.23 ± 0.56, and 39.74 ± 0.42%IACS, respectively. These standard deviations represent ten spatial measurements on one specimen per composition. The narrow range supports retention of a continuous Cu-rich conduction pathway, but the measurements do not quantify three-dimensional Cu connectivity directly.

At 500 °C, the thermal conductivities of Cu–(CrW0)C, Cu–(CrW10)C, Cu–(CrW25)C, and Cu–(CrW50)C are 172.49 ± 0.40, 208.27 ± 0.29, 184.95 ± 0.24, and 183.55 ± 0.37 W m^−1^ K^−1^, respectively. The error bars describe three repeated laser-flash shots on one specimen at each temperature. Cu–(CrW10)C shows the highest *λ*_500_, whereas the non-monotonic trend indicates that Cu-path continuity, carbide-skeleton topology, and Cu/carbide interfacial scattering jointly affect thermal transport.

The thermal-expansion curves referenced to 30 °C increase continuously with temperature. The average CTE values from 30 to 500 °C are 13.97, 13.98, 13.23, and 13.36 × 10^−6^ K^−1^ for the 0 W, 10 W, 25 W, and 50 W IPCs, respectively. One dilatometry specimen was measured per composition; therefore, the CTE values are not accompanied by specimen-to-specimen error bars.

To further compare the measured thermal conductivity with the electronic transport capability estimated from electrical conductivity, a transport consistency factor, *η*_TC_, was introduced. A value close to 100% indicates that the measured room-temperature thermal conductivity approaches the electronic thermal conductivity estimated from the corrected electrical conductivity, confirming the dominant contribution of the continuous Cu-rich network to heat and charge transport. The deviation from 100% reflects additional thermal-transport losses associated with Cu/carbide interfaces, skeleton topology, and W-rich heterogeneity. These results provide the thermophysical and electrical basis for analyzing the subsequent thermo-tribological response.

### 3.6. Friction Coefficient and Quantitative Wear Resistance

**Figure 6:**
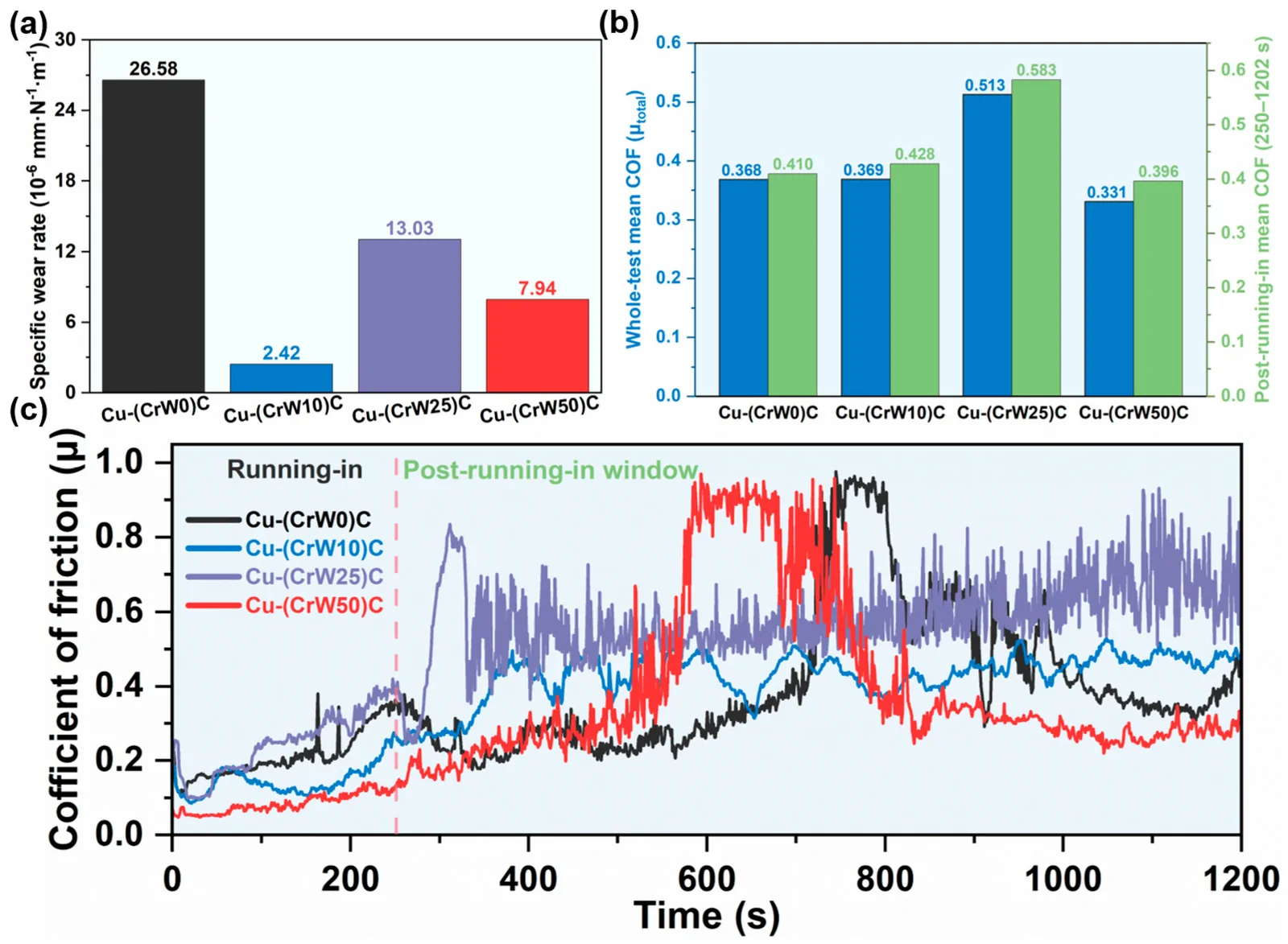
Friction and wear behavior of Cu–(CrW*_x_*)C IPCs: (**a**) specific wear rate; (**b**) whole-test and post-running-in (250–1202 s) mean coefficients of friction (COFs); and (**c**) complete COF-time curves. The fixed post-running-in window does not imply strict stationarity.

The friction and wear results are shown in Figure 6. The whole-test mean COFs of Cu–(CrW0)C, Cu–(CrW10)C, Cu–(CrW25)C, and Cu–(CrW50)C are 0.368, 0.369, 0.513, and 0.331, respectively; the corresponding fixed-window means over 250–1202 s are 0.410, 0.428, 0.583, and 0.396. The latter values are reported as post-running-in comparison means rather than steady-state coefficients because pronounced transitions occur after 250 s, including a high-friction event at approximately 722–813 s for Cu–(CrW0)C and a broad transition at approximately 571–762 s for Cu–(CrW50)C. Cu–(CrW10)C nevertheless exhibits the lowest specific wear rate, 2.42 × 10^−6^ mm^3^ N^−1^ m^−1^, compared with 26.58, 13.03, and 7.94 × 10^−6^ mm^3^ N^−1^ m^−1^ for the 0 W, 25 W, and 50 W IPCs. Thus, low friction and low material loss are not equivalent in this short-duration test.

### 3.7. Worn Surface Morphology

**Figure 7:**
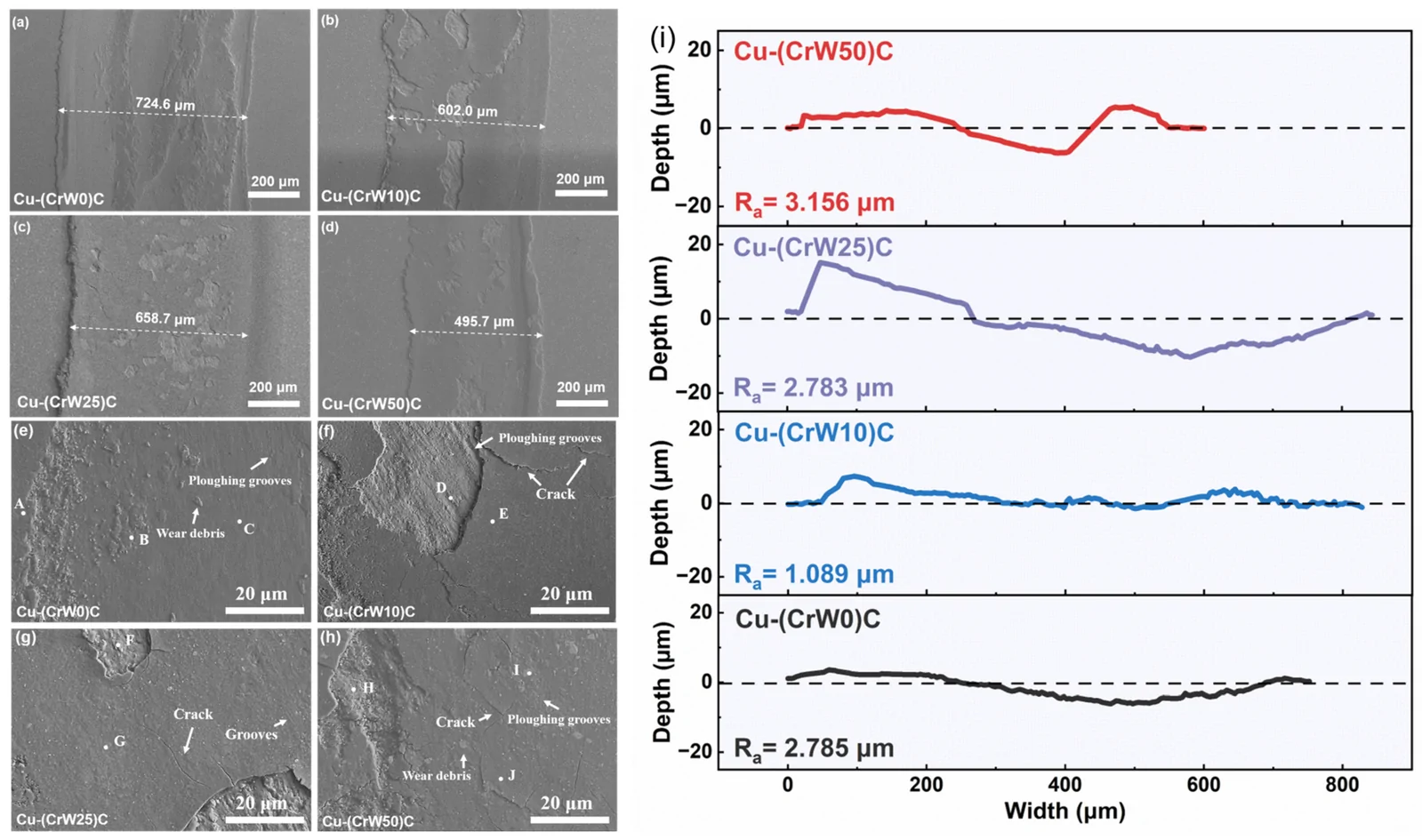
Worn surface morphologies of Cu–(CrW*_x_*)C IPCs after sliding: (**a**–**d**) low-magnification SEM images; (**e**–**h**) local high-magnification morphologies; and (**i**) representative transverse wear-scar profiles, arranged from top to bottom as Cu–(CrW50)C, Cu–(CrW25)C, Cu–(CrW10)C, and Cu–(CrW0)C. Uppercase labels A–J denote the EDS point-analysis locations reported in Table S1 of the Supplementary Materials.

The worn surface morphologies are shown in Figure 7. The wear scar widths of Cu–(CrW0)C, Cu–(CrW10)C, Cu–(CrW25)C, and Cu–(CrW50)C are 724.6, 602.0, 658.7, and 495.7 μm, respectively, whereas their integrated wear volumes are 0.056207, 0.005120, 0.027554, and 0.016781 mm^3^. The narrowest scar therefore does not correspond to the lowest material loss. Cu–(CrW0)C shows grooves, roughened regions, and localized delamination-like damage. Cu–(CrW10)C contains localized cracking and sheet-like adhered or partially detached features; its superior wear resistance is supported by the low integrated wear volume rather than by an absence of visible surface damage. Cu–(CrW25)C exhibits fragmented/flaky regions and grooves, while Cu–(CrW50)C remains locally cracked and debris-covered despite its narrower scar. The transverse profiles in Figure 7i are interpreted together with the integrated three-dimensional wear volumes and are not used as direct substitutes for total material loss.

### 3.8. EDS Mapping of Worn Surfaces

**Figure 8:**
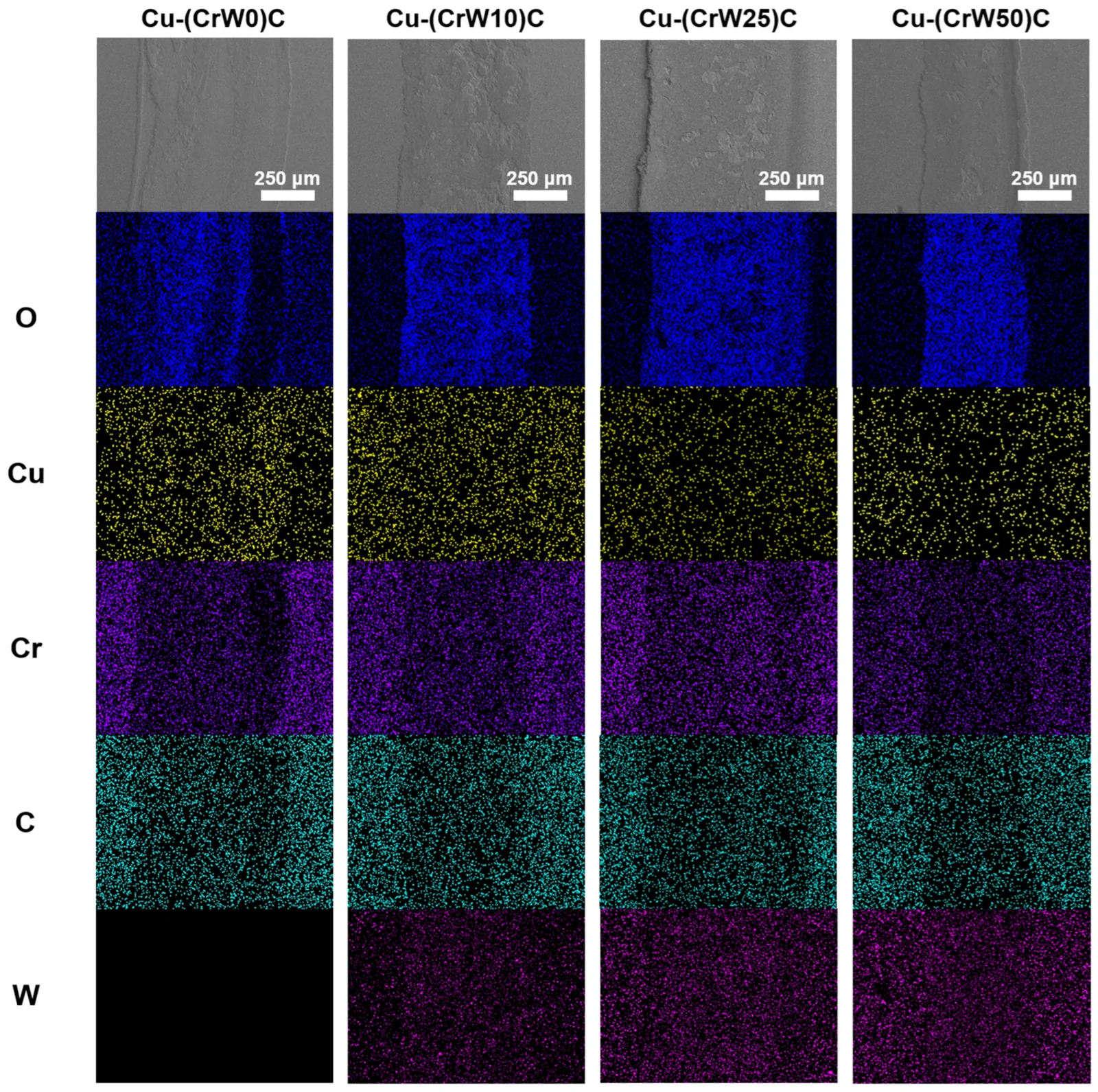
Worn surface SEM images and corresponding EDS maps of Cu–(CrW*_x_*)C IPCs. Columns from left to right correspond to Cu–(CrW0)C, Cu–(CrW10)C, Cu–(CrW25)C, and Cu–(CrW50)C; rows from top to bottom are SEM, O, Cu, Cr, C, and W. All maps were acquired and displayed using identical conditions and are interpreted qualitatively. Point-analysis values are reported in Table S1.

EDS mapping of the worn surfaces is shown in Figure 8. All four datasets were acquired and displayed using identical conditions. Cu, Cr, C, and O signals are heterogeneously distributed across the worn regions, indicating a tribologically modified surface containing exposed metallic and carbide regions, compacted debris, and localized oxidation products. The maps do not demonstrate a continuous Cu-rich oxide film, and brightness is not converted into composition. Location-specific point analyses in Table S1 provide the stronger evidence: A and D are locally Cu–O enriched; E, G, H, and J are multicomponent oxygen-rich regions; B, C, and F are Cr–C-dominated carbide regions; and point I in Cu–(CrW50)C contains 12.3 at.% W with only 1.5 at.% Cu, supporting localized W-rich carbide/debris exposure. The absence of detectable W at selected 10 W points does not imply that the bulk 10 W skeleton contains no W.

### 3.9. Cross-Sectional Damage Beneath Worn Scars

**Figure 9:**
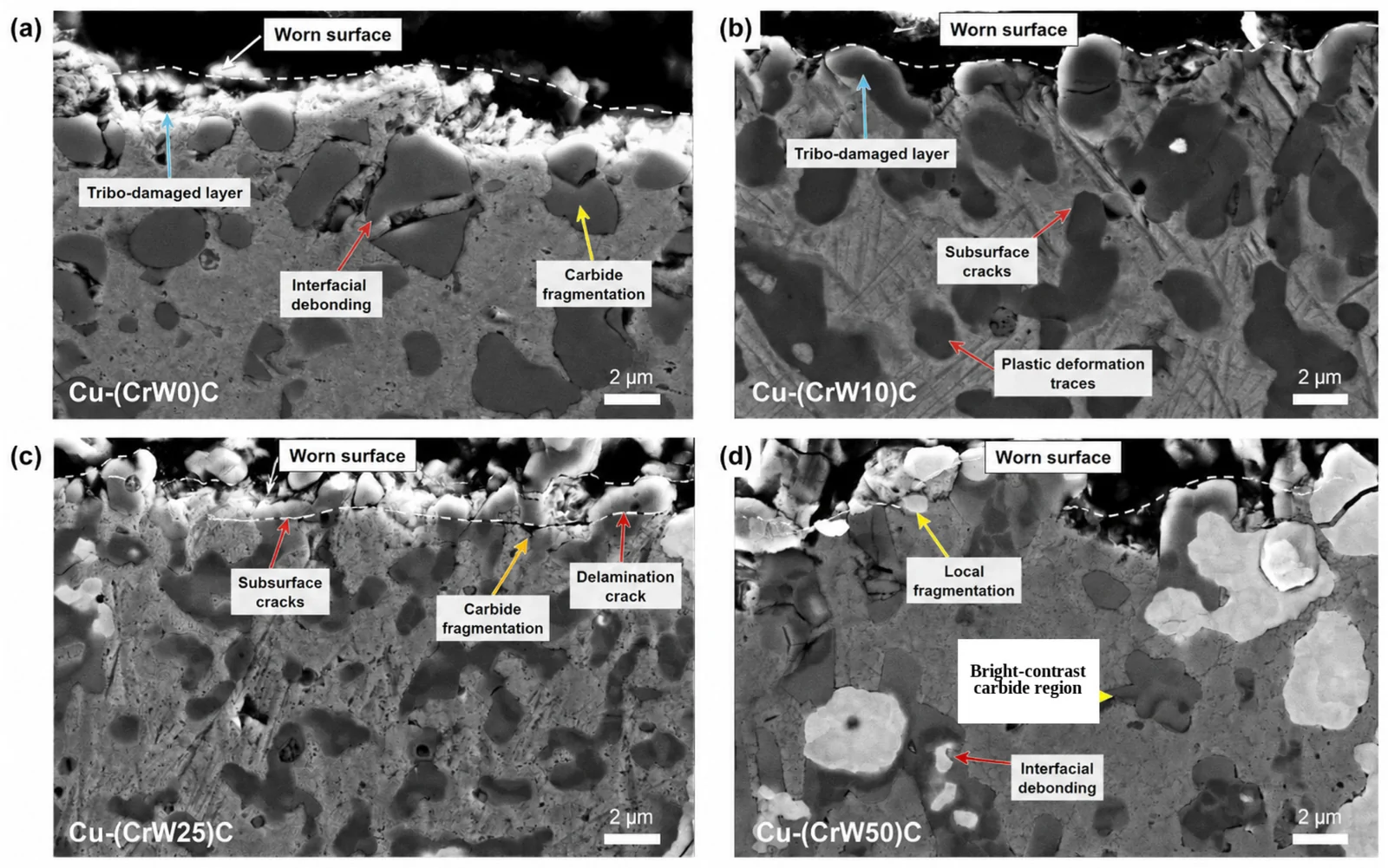
Cross-sectional SEM images beneath the worn scars of (**a**) Cu–(CrW0)C, (**b**) Cu–(CrW10)C, (**c**) Cu–(CrW25)C, and (**d**) Cu–(CrW50)C. Labels identify directly observed features, including worn surfaces, tribologically damaged regions, subsurface cracks, carbide fragmentation, plastic deformation traces, interfacial separation, and a bright-contrast carbide region. No cross-sectional W-rich assignment is made without location-matched EDS.

Cross-sectional SEM observations clarify subsurface damage beneath the wear scars (Figure 9). Cu–(CrW0)C shows carbide fragmentation, interfacial separation, and localized near-surface material loss. Cu–(CrW10)C still contains localized subsurface cracks and plastic deformation traces; its lower integrated wear volume is consistent with less extensive net removal rather than a crack-free subsurface. Cu–(CrW25)C exhibits fragmented carbide, pores/microcracks, and broader damage. Cu–(CrW50)C shows local fragmentation and interfacial separation together with bright high atomic number contrast carbide regions. Because no location-matched cross-sectional EDS was acquired, these bright regions are not labeled as W-rich skeletons. Surface point I demonstrates local W enrichment on the worn surface but cannot be transferred to a different cross-sectional location.

### 3.10. Quantitative Thermo-Tribological Coupling Analysis

The key thermo-tribological coupling indicators calculated from frictional work, wear volume, thermal conductivity, and thermal expansion are summarized in Table 1. Cu–(CrW10)C exhibits the lowest specific wear rate and the lowest wear loss per unit frictional work, indicating that material removal under a given frictional energy input is effectively suppressed. It also possesses the highest thermal conductivity at 500 °C while maintaining an acceptable *α*_30–500_. Consequently, Cu–(CrW10)C achieves the highest normalized TTPI, confirming the most favorable balance among frictional damage resistance, heat dissipation, and thermal dimensional stability. Hardness and the quasi-static compression response are used to interpret mechanical support and resistance to local deformation, whereas electrical conductivity is used to verify the continuity of the Cu-rich transport network. These parameters are not directly included in the TTPI expression, which avoids reducing the IPC response to a single mechanical or transport parameter and emphasizes that the optimum composition must satisfy multiple constraints simultaneously.

**Figure 10:**
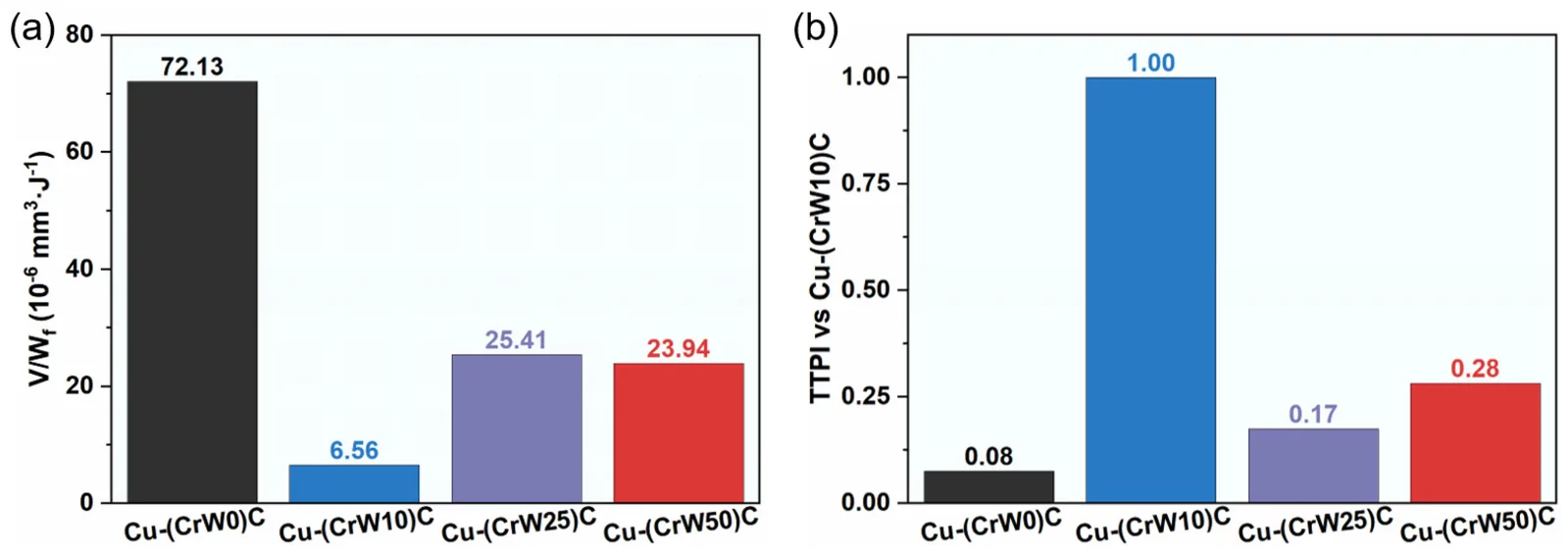
Derived thermo-tribological indicators of Cu–(CrW*_x_*)C IPCs: (**a**) wear loss per unit frictional work, *K*_E_ = *V*/*W*_f_, where *V* is wear volume and *W*_f_ is frictional work; and (**b**) thermo-tribological performance index (TTPI) normalized to Cu–(CrW10)C. The measured input values and other derived parameters are reported in Table 1.

Based on the parameters listed in Table 1, the thermo-tribological coupling performance is visualized in Figure 10. Cu–(CrW10)C combines the lowest wear rate, the lowest wear loss per unit frictional work, the highest thermal conductivity at 500 °C, and the highest normalized TTPI. These results indicate that its superior performance is not derived from a low friction coefficient, high hardness, or high compressive load-bearing capacity alone, but from an optimized balance among friction-induced damage resistance, heat dissipation, thermal expansion stability, and skeleton/Cu-network cooperation. The following performance-positioning analysis further compares this balanced response with reported W–Cu-based wear-resistant materials and highlights the electrical-thermal-wear balance within the present IPC series. This result identifies Cu–(CrW10)C as the composition with the most favorable thermo-tribological balance, rather than as a material optimized for a single property.

### 3.11. Performance Positioning and Electrical-Thermal-Wear Balance

**Figure 11:**
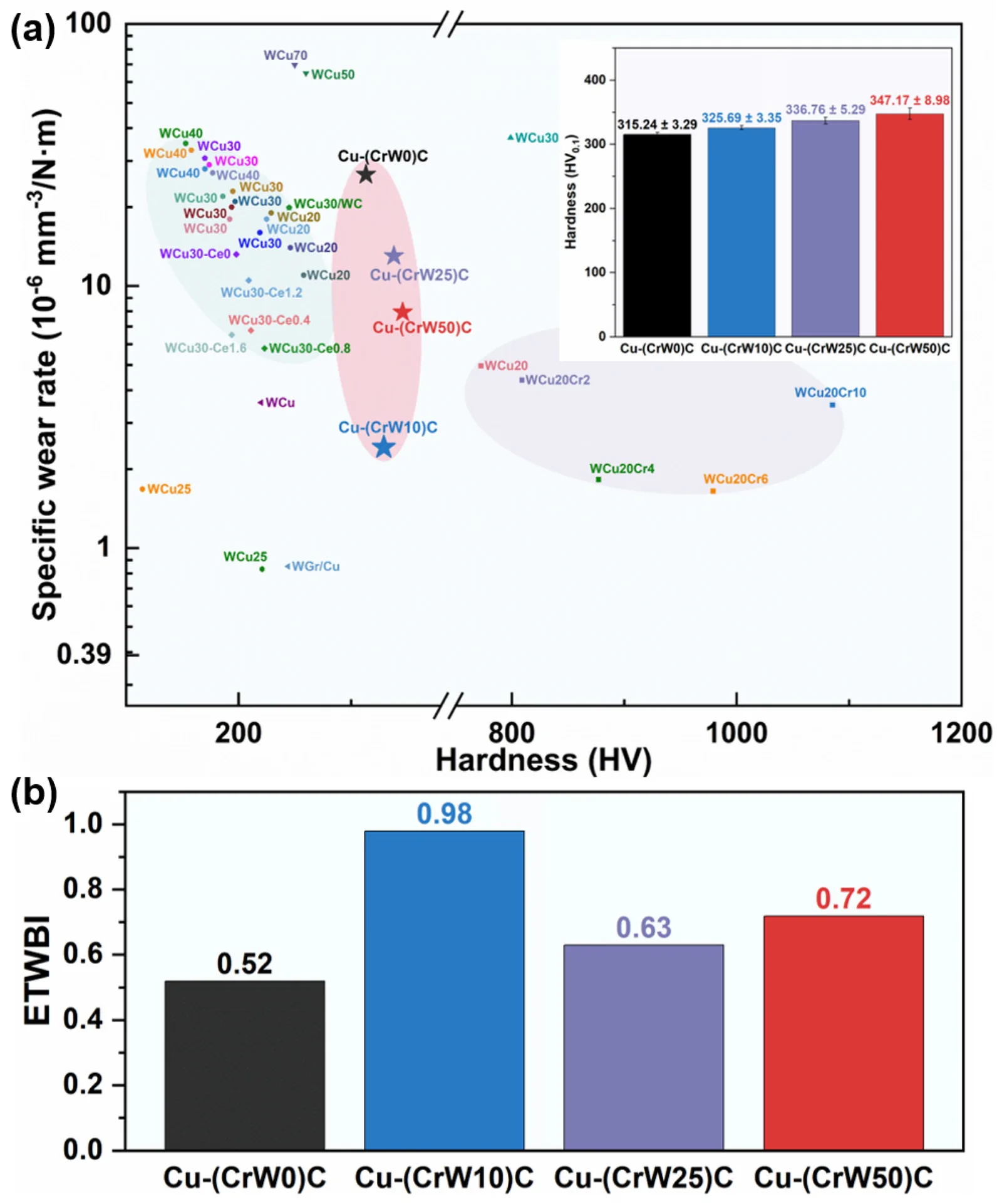
Performance positioning and electrical-thermal-wear balance of Cu–(CrW*_x_*)C IPCs: (**a**) hardness-specific wear-rate map with the inset showing the mean ± SD of Vickers microhardness measured with a 0.1 kgf test force (HV0.1) from ten indentations on one specimen; (**b**) recalculated electrical-thermal-wear balance index (ETWBI) values of the present IPCs. Literature data in (**a**) correspond to Refs. [35,36,37,38,39,40,41] and are shown for positioning rather than direct ranking. Symbols in (**a**): ★, present IPCs; ●, Ref. [35]; ■, Ref. [36]; ⬟, Ref. [37]; ◆, Ref. [38]; ▲/▼, Ref. [39]; ⬢, Ref. [40]; and ◀, Ref. [41].

The hardness-specific wear rate map in Figure 11a is used for performance positioning rather than direct ranking because the literature wear rates were obtained under different test conditions. The HV0.1 values of Cu–(CrW0)C, Cu–(CrW10)C, Cu–(CrW25)C, and Cu–(CrW50)C are 315.24 ± 3.29, 325.69 ± 3.35, 336.76 ± 5.29, and 347.17 ± 8.98, respectively, based on ten indentations on one specimen per composition. Hardness increases with nominal W addition, whereas the specific wear rate remains non-monotonic. Cu–(CrW10)C therefore has the lowest material loss despite not having the highest hardness, supporting the role of damage-layer stability and debris control [35,36,37,38,39,40,41].

The recalculated ETWBI values are 0.52, 0.98, 0.63, and 0.72 for Cu–(CrW0)C, Cu–(CrW10)C, Cu–(CrW25)C, and Cu–(CrW50)C, respectively. The ranking is unchanged: Cu–(CrW10)C > Cu–(CrW50)C > Cu–(CrW25)C > Cu–(CrW0)C. The 10 W advantage arises from the combined high *λ*_500_, retained electrical conductivity, low specific wear rate, and acceptable CTE rather than from maximizing a single property. Because ETWBI uses dataset-dependent normalization and equal weighting, its values are confined to internal comparison of the present four compositions.

## 4. Conclusions

Cu–(CrW*_x_*)C IPCs were fabricated using nominal W-addition indices of 0, 10, 25, and 50 g W per 100 g Cr_2_O_3_. W addition changed the scale, local constitution, and connectivity of W-containing carbide regions, while the estimated Cu-phase volume fraction remained within 55.68–56.84 vol.% and the continuous Cu-rich network maintained electrical conductivities of 39.23–40.44%IACS. The reconstructed 39–41° XRD peak group is compatible with compositionally heterogeneous W-substituted Cr_3_C_2_-type regions, but the present laboratory data do not permit a unique phase assignment, W-substitution level, or quantitative phase fraction. The narrow Cu-fraction range further indicates that the composition-dependent properties are governed mainly by architectural and interfacial changes rather than by the total Cu content alone.

Cu–(CrW10)C exhibited *λ*_500_ = 208.27 W m^−1^ K^−1^ and the lowest specific wear rate, 2.42 × 10^−6^ mm^3^ N^−1^ m^−1^. Its worn surface was not crack-free; the low material loss instead reflects a favorable balance among fine W-containing features, Cu-phase plastic/thermal accommodation, and limited deep material removal. Higher nominal W additions increased hardness but also promoted carbide fragmentation, interfacial separation, and hard debris-mediated abrasion. TTPI and ETWBI identify the 10 W IPC as the most balanced composition within this dataset, but these indices are internal screening tools rather than universal properties.

The compression curves are representative and do not establish specimen-to-specimen statistical differences. Likewise, the 1202 s wear tests represent one load speed-counterbody condition and do not establish long-term electro-tribological stability. Independent compression replicates, longer duration wear tests, and coupled electrical contact experiments are required before application-level extrapolation.

## Figures and Tables

**Table 1:** Measured inputs and derived thermo-tribological indicators of Cu–(CrW*_x_*)C interpenetrating phase composites (IPCs).

Sample	*K*	*K*_E_	*λ*_500_ Mean ± SD	*λ*_500_ RMSD	*α*_30–500_	*H*	*Π*_th_	TTPI
Cu–(CrW0)C	26.58	72.13	172.49 ± 0.40	0.33	13.97	3.76	52.51	0.08
Cu–(CrW10)C	2.42	6.56	208.27 ± 0.29	0.24	13.98	3.12	43.61	1.00
Cu–(CrW25)C	13.03	25.41	184.95 ± 0.24	0.20	13.23	4.88	64.52	0.17
Cu–(CrW50)C	7.94	23.94	183.55 ± 0.37	0.30	13.36	3.18	42.45	0.28

Notes: *K* denotes the specific wear rate; *K*_E_ denotes wear loss per unit frictional work; *λ*_500_ denotes thermal conductivity at 500 °C; *α*_30–500_ denotes the average coefficient of thermal expansion from 30 to 500 °C; *H* denotes the relative heat-accumulation tendency; *Π*_th_ denotes the thermal-stress tendency; and TTPI denotes the thermo-tribological performance index. *K* is reported in 10^−6^ mm^3^ N^−1^ m^−1^; *K*_E_ in 10^−6^ mm^3^ J^−1^; *λ*_500_ in W m^−1^ K^−1^; *α*_30–500_ in 10^−6^ K^−1^; *H* and *Π*_th_ as 10^−3^. Thermal conductivity standard deviation (SD) and root-mean-square deviation (RMSD) are calculated from three repeated shots on one specimen at 500 °C. *K* is based on one integrated wear volume measurement per composition; no specimen-to-specimen wear dispersion is claimed. TTPI is normalized to Cu–(CrW10)C.

## Data Availability

The original contributions presented in this study are included in the article/Supplementary Materials. Further inquiries can be directed to the corresponding authors.

## Supplementary Materials

The following supporting information can be downloaded at: https://www.mdpi.com/article/10.3390/ma19163394/s1, Table S1. EDS point-analysis results for representative locations A–J on the worn surfaces of Cu-(CrW*_x_*)C (*x* = 0, 10, 25, and 50) IPCs (see Figure 7 in the main text). Table S2. Mean values and root-mean-square deviation (RMSD) of thermal conductivities measured at 25–500 °C for Cu-(CrW*_x_*)C (*x* = 0, 10, 25, and 50) interpenetrating phase composites.
