# Supplementary material for: Balancing Thermal Management and Wear Resistance via Tungsten-Mediated Architectural Stabilization of Cu-Based Interpenetrating Phase Composites
Source: Materials (Basel). 2026 Aug 10;19(16):3394. doi: 10.3390/ma19163394 (PMC13514816; doi:10.3390/ma19163394)
Supplement: Supplementary file 1 [file materials-19-03394-s001.zip › materials-4462157-supplementary.pdf]

Supplementary Materials of

Balancing Thermal Management and Wear Resistance via Tungsten-Mediated Architectural Stabilization of Cu-Based Interpenetrating Phase Composites

Han Hu [1], Ziqiang Dong [1*], Yanjie Liu [2*] and Yi Liu [1,3*]

[1] Materials Genome Institute & State Key Laboratory of Materials for Advanced Nuclear Energy & Shanghai Engineering Research Center for Integrated Circuits and Advanced Display Materials, Shanghai University, Shanghai 200444, China; hanhu0526@shu.edu.cn (H. H.)
[2] School of Mechanical and Energy Engineering, Shanghai Technical Institute of Electronics & Information, Shanghai 201411, China
[3] Institute of Low-Dimensional Carbons and Device Physics, Shanghai University, Shanghai 200444, China

[*]Correspondence: zqdong@shu.edu.cn (Z.D.); hnlylyj@163.com (Yanjie Liu); yiliu@shu.edu.cn (Yi Liu)

Table S1. Energy-dispersive X-ray spectroscopy (EDS) point-analysis results for representative locations A–J on the worn surfaces of Cu-(CrW$_x$)C (x = 0, 10, 25, and 50) interpenetrating phase composites (IPCs) (see Figure 7 in the main text).

Point	Cr	Cu	C	O	W	Si
A	3.0	45.6	27.7	22.6	–	1.2
B	37.2	6.3	56.5	–	–	–
C	33.1	10.4	49.9	6.6	–	–
D	12.2	39.9	23.4	23.8	–	0.7
E	16.1	19.3	22.6	39.2	–	2.7
F	23.2	17.5	50.4	8.4	0.6	–
G	17.1	24.6	25.1	31.5	0.5	1.3
H	3.6	14.5	35.4	43.1	1.4	2.0
I	12.7	1.5	66.6	6.8	12.3	–
J	10.0	13.3	40.4	33.3	1.4	1.7

Notes: Values are reported in atomic percent (at.%). "–" indicates not detected or below the quantitative detection limit. Carbon contents are semiquantitative and are not used to determine exact carbide stoichiometry. Minor Si at selected oxygen-containing points may be associated with counterbody transfer, but no specific Si-containing phase is assigned.

Table S2. Mean values and root-mean-square deviation (RMSD) of thermal conductivities measured at 25–500 °C for Cu-(CrW$_x$)C (x = 0, 10, 25, and 50) interpenetrating phase composites.

Sample	Temperature (°C)	Mean (W m^{-1} K^{-1})	RMSD
Cu-(CrW0)C	25	170.84	0.33
Cu-(CrW0)C	300	168.50	0.45
Cu-(CrW0)C	500	172.49	0.33
Cu-(CrW10)C	25	161.19	0.09
Cu-(CrW10)C	300	186.56	0.35
Cu-(CrW10)C	500	208.27	0.24
Cu-(CrW25)C	25	160.44	0.17
Cu-(CrW25)C	300	169.12	0.29
Cu-(CrW25)C	500	184.95	0.20
Cu-(CrW50)C	25	155.22	0.53
Cu-(CrW50)C	300	169.02	0.27
Cu-(CrW50)C	500	183.55	0.30

Notes: At each temperature, three repeated laser-flash shots were collected on one specimen. RMSD is the root-mean-square deviation about the three-shot mean. These values quantify repeated-shot measurement dispersion rather than specimen-to-specimen variability.
